# Single‐cell transcriptomic landscape reveals the differences in cell differentiation and immune microenvironment of papillary thyroid carcinoma between genders

**DOI:** 10.1186/s13578-021-00549-w

**Published:** 2021-02-15

**Authors:** Miaoguan Peng, Guohong Wei, Yunjian Zhang, Hai Li, Yingrong Lai, Yan Guo, Yuxin Chen, Liehua Liu, Haipeng Xiao, Hongyu Guan, Yanbing Li

**Affiliations:** 1 Department of Endocrinology and Diabetes Center, The First Affiliated Hospital of Sun Yat-sen University, 58 Zhongshan Road II, Guangdong 510080 Guangzhou, China; 2 Department of Thyroid and Breast Surgery, The First Affiliated Hospital of Sun Yat-sen University, 58 Zhongshan Road II, Guangdong 510080 Guangzhou, China; 3 Department of Pathology, The First Affiliated Hospital of Sun Yat-sen University, 58 Zhongshan Road II, Guangdong 510080 Guangzhou, China

**Keywords:** Papillary thyroid carcinoma, Single‐cell RNA-sequencing, Cell differentiation, Immune microenvironment, Gender‐driven

## Abstract

**Background:**

Papillary thyroid carcinoma (PTC) is the main pathological type of thyroid carcinoma (TC). Gender is a prominent background parameter for patients with PTC. Here, we aimed to delineate the differences in cell clusters and immune microenvironment in relation to gender in PTC.

**Methods:**

We generated 6720, 14,666, and 33,373 single-cell transcriptomes that were pooled from the tissues of four male patients with PTC, seven female patients with PTC, and three patients with nodular goiter, respectively. We performed single-cell RNA-sequencing (scRNA-seq) based on BD Rhapsody and characterized the first single-cell transcriptomic landscape of PTC involving gender. The differential cell clusters and their gene profiles were identified and analyzed via a multi-resolution network in male and female patients. The interactions of fibroblasts and endothelial cells with malignant epithelial cells and the difference in the immune infiltration of B and T lymphocytes according to gender were assessed.

**Results:**

Malignant epithelial cells were divided into two distinct subsets in male and female patients with PTC. Moreover, significant differences involving inferred copy-number variations (CNVs), gene profiles, and cell differentiation were detected between male and female patients. Regarding the interactions of fibroblasts and endothelial cells with malignant epithelial cells, members of the human leukocyte antigen (*HLA*) family and their receptors were considered as typical in female patients with PTC, while transforming growth factor beta 1 (TGFB1) and its receptors were typical of male patients with PTC. The characteristics of B cells, including cell clusters, cell differentiation, and dominant gene sets, were significantly different between genders.

**Conclusions:**

Our data revealed the detailed differences in cell clusters and immune microenvironment in PTC according to gender at the single-cell level, which provided new insights into the understanding of the impact of gender on PTC.

## Introduction

Thyroid cancer is the most common endocrine-system malignancy worldwide [1, 2]. PTC is the most common histopathological type of TC, accounting for about 75–85% [3]. Intriguingly, obvious differences were observed between male and female patients [1, 4–6]. The incidence of PTC in women is significantly higher than that in men and the female-to-male ratio is about three or more [1, 4]. Moreover, epidemiological data indicate that male patients with PTC have a more aggressive phenotype and worse clinical outcomes [6]. Although females exhibit a higher incidence of PTC, male gender has been shown to be associated with higher rates of advanced disease stage and a worse disease prognosis. Kurozumi et al. showed that extrathyroidal extension was closely related to male gender [7]. Machens and colleagues performed a retrospective cohort analysis and the results showed that the primary tumor size of PTC in male patients was significantly larger than that in female patients and male patients had a higher incidence of lymph node metastases and distant metastases [8]. Nevertheless, the differences induced by gender in PTC are little understood at the cellular level.

The tumor immune microenvironment (TIME) is a collection of immune cells and related immune factors (including innate and adaptive immune factors) located in and around tumor tissues [9]. The interaction between malignant cells and the proximal immune cells leads to an environment that promotes tumor growth and metastasis [10, 11]. The therapeutic strategies targeting the TIME are expected to be highly promising in view of the powerful regulatory ability of the TIME on tumor cells [12–15]. Single-cell transcriptomic sequencing is a technology that developed rapidly in the last decade that can accurately distinguish and explore cell clusters and related gene profiles in complex mixtures [16, 17].

Here, the single-cell transcriptomic landscape of PTC was characterized for the first time and the differences in cell clusters between male and female patients with PTC were identified. We also analyzed the interactions of fibroblasts and endothelial cells with malignant epithelial cells, as well as the differences in the immune infiltration of B and T lymphocytes.

## Methods

### Patients and demographic data

Human samples of either nodular goiter or PTC were collected from patients who received surgery at the First Affiliated Hospital of Sun Yat-sen University (Guangzhou, China) from March 2020 to April 2020. All patients were diagnosed with the corresponding disease types, and informed consent was obtained before sample collection. Patients with autoimmune thyroid disease (Hashimoto’s thyroiditis or lymphocytic thyroiditis) were excluded. In total, fourteen patients (three females with nodular goiter, seven females with PTC, and four males with PTC) were enrolled in the study. Age of males with PTC averaged 38.0 years with a range of 30–50 years. Age of females with PTC averaged 36.6 years with a range of 20–70 years. Age of patients with nodule averaged 39.0 years with a range of 29–56 years. Tumor size of male averaged 1.53 cm with a range of 0.5–3.0 cm and female averaged 1.04 cm with a range of 0.3–1.2 cm. Nodule size of averaged 2.43 cm with a range of 0.4–4.6 cm. There were no multicentricity, extrathyroidal invasion and distant metastasis in PTC. Almost all the patients with PTC at TNM stage I except one male with PTC at stage II. All the above operations were in accordance with the relevant ethical norms and were approved by the Institutional Research Ethics Committee.

### Preparation of single‐cell samples

Human tissues were rinsed twice with cold saline and then minced using scissors. Tissue fragments were collected for enzymatic digestion in Dulbecco’s Modified Eagle’s Medium (DMEM, Gibco, Gaithersburg, MD, USA) containing 40 U/ml DNase type I (Gibco, Carlsbad, CA, USA) and 1.0 ml/ml collagenase type IV (Sigma-Aldrich, St. Louis, Missouri, USA). The digestive system was incubated at 37 °C for 1 h, and cells were then passed through a 70 µm nylon cell strainer (Millipore, Billerica, MA, USA). The cells were centrifuged and resuspended with fetal bovine serum (FBS, Gibco, Grand Island, NY, USA). The prior viability and cell density were detected by trypan blue, and the average viability rate of cells before loading was no less than 90%. We loaded PTC cells from male patients into one channel, cells from female patients into a second channel and cells from patients with goiter into the third channel. Each of these three pooled samples was prepared for the next step of single-cell library preparation.

### Single‐cell RNA-sequencing

We performed single-cell RNA-sequencing (scRNA-seq) based on BD Rhapsody and characterized the first single-cell transcriptomic landscape of PTC involving gender. Single-cell libraries were prepared using the BD Rhapsody Single-Cell Analysis System (BD, USA), according to the manufacturer’s protocol. Libraries were sequenced using multiple runs on an Illumina NextSeq platform. The differential cell clusters and their gene profiles were identified and analyzed via a multi-resolution network in male and female patients. The interactions of fibroblasts and endothelial cells with malignant epithelial cells and the difference in the immune infiltration of B as well as T lymphocytes according to gender were assessed.

### Unsupervised clustering of cells and *t*-distributed Stochastic Neighbor Embedding (tSNE) visualization

The Seurat package (version 3.1.2) implemented in R was used to perform the unsupervised clustering of cells. Genes that were expressed in less than two cells were filtered out. Cells with expression of > 200 genes and < 10% mitochondrial genes were further processed. The variation coefficient of genes was calculated using the Seurat arithmetic. Dimensionality reduction of all data was performed by principle component analysis (PCA) according to the first 1500 highest alterable genes. A k-nearest neighbor graph was executed with Euclidean distances in the space of the first 10 significant principal components. The cells were clustered by the Louvain Modularity optimization algorithm and visualized by tSNE visualization.

### Marker gene identification and cell annotation

To ascertain marker genes, the single-cell gene expression of each cluster was calculated using the “bimod” test implemented in the Seurat Find Markers function. Only genes with an expression with a log2 average difference of 0.585 and *P* < 0.05 were classified as marker genes. ImmGen and Enrichr were used to represent cell clusters, which were annotated by canonical markers of known cell types.

### Infer CNV analysis

According to the mRNA copy detected by single-cell sequencing, the gene was located on the genome, and the heat map of inferred CNV was drawn. Taking the nodular goiter group as the reference, the differences were analyzed according to the copy number of mRNA and the degree of sequence variation, and the tumor cells were identified.

### Hallmark signal pathway analysis

The hallmark gene sets were obtained from the public Molecular Signature Database, and the signal pathway activity of each cell was scored using gene set variation analysis methods, according to a standardized strategy. Student’s *t*-test was used to calculate the differences in pathways between two groups. Significance was set at *P* < 0.05.

### KEGG enrichment analysis

KEGG analysis of selected genes was performed using the KOBAS software, with Benjamini-Hochberg testing adjustments.

### Statistical analysis of population shifts

The significant alteration of the percent composition of cell clusters in samples was evaluated using the Benjamini-Hochberg correction and the nonparametric Kruskal-Wallis test with Dunn’s multiple comparisons test.

### Data statistics

Data are presented as the mean ± SEM, and differential analysis was performed via either variance (three groups) or Student’s *t*-test (two groups) using GraphPad Prism 7 Significance was set at *P* < 0.05.

## Results

### Determination of the human PTC transcriptomic landscape

**Fig. 1:**
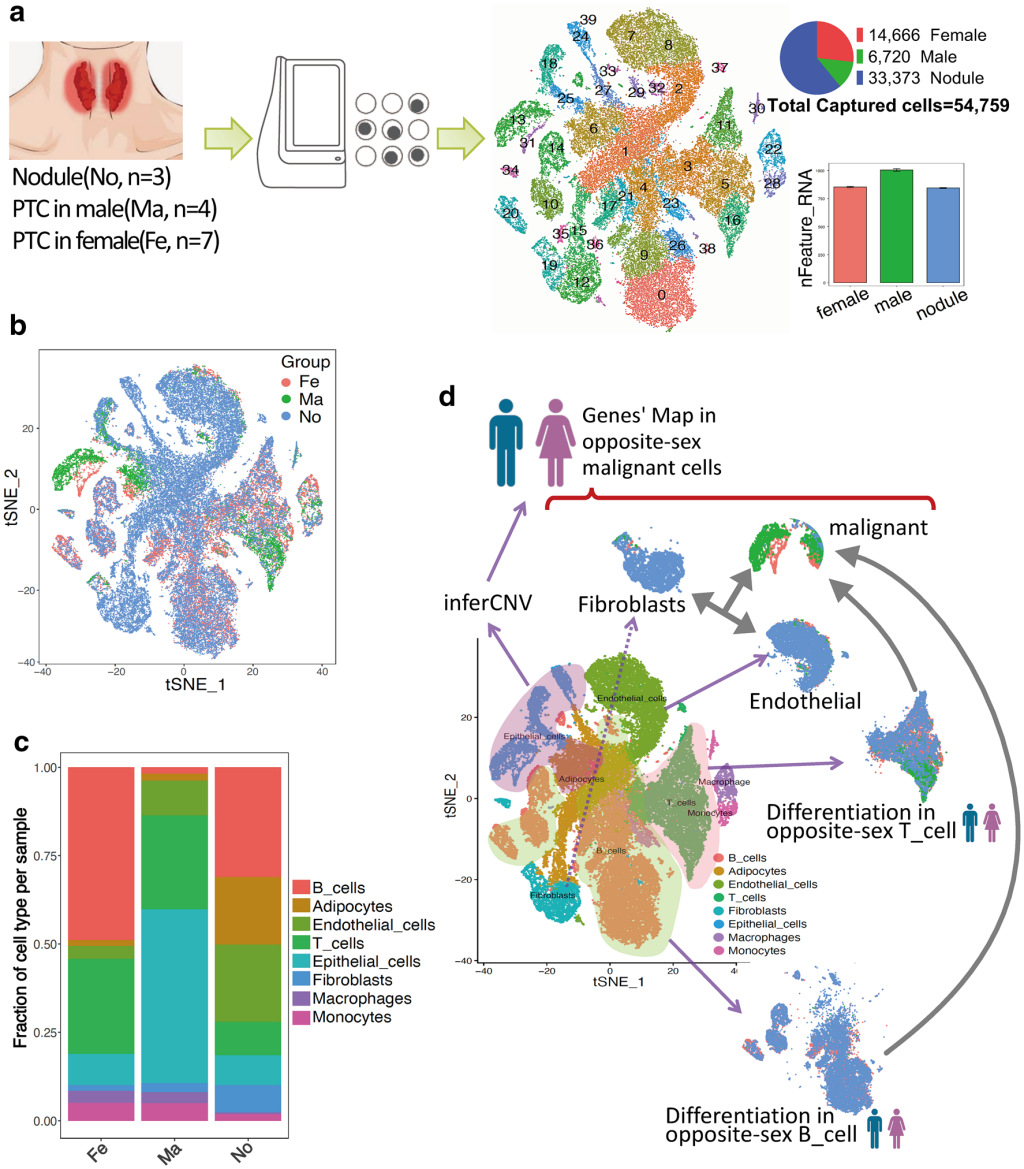
Transcriptome profiling of the three groups at the single-cell level. **a** Process and principle of the experiment. Tissues were collected and isolated for the preparation of single-cell libraries, which were then sequenced and all data were analyzed. **b** tSNE plot visualization showed cell fractions in three groups. The subjects of the three groups are displayed using different colors. **c** The frequency of representative cell clusters in the three groups is shown in the bar graph. **d** Subsequent data analysis strategy for inferCNV, including gene mapping in opposite-sex malignant cells and differentiation in opposite-sex T and B cells

Cells were isolated respectively from three patients with nodular goiter, four male patients with PTC, and seven female patients with PTC, followed by single-cell transcriptomic sequencing based on BD Rhapsody (Fig. 1a). All cell clusters were differentiated according to the three disease statuses (nodular, male PTC, and female PTC), which showed that the dominant clusters were different between male and female patients (Fig. 1b). The proportion of endothelial cells, fibroblasts, and B cells in female PTC patients were greatly higher than these in male PTC patients (Fig. 1c). Next, we focused on malignant epithelial cells, fibroblasts, endothelial cells, T cells and B cells by analyzing the differences in cell differentiation and gene expression profiles, as well as their interactions according to gender (Fig. 1d).

### Differences in cell differentiation and gene profiles of malignant epithelial cells between male and female patients with PTC

**Fig. 2:**
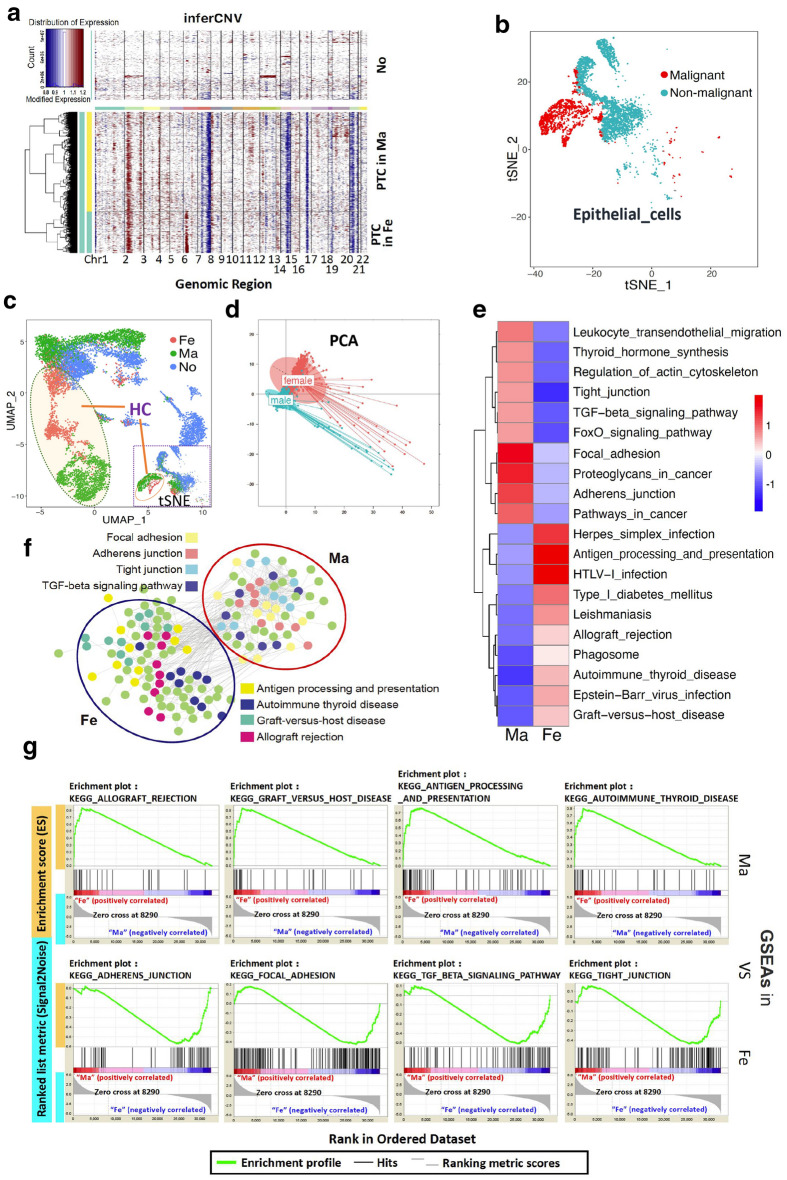
Cell clusters and gene expression of malignant epithelial cells in male- and female patients. **a** The gene copy-number variation of epithelial cells in male and female patients with PTC and in the nodular goiter group was analyzed using the infer CNV algorithm. **b** Two-dimensional tSNE visualization showed the malignant and non-malignant epithelial cells in the three groups. The subjects with either malignant or non-malignant cells are displayed using different colors. **c** The distribution of epithelial cells in the three groups was marked in the total epithelial cells in the tSNE visualization (Right) (“HC” is the abbreviation of “Heterogeneous clusters”). The heterogeneous distribution of malignant epithelial cells in male and female patients with PTC was analyzed via UMAP (Left). **d** PCA of malignant epithelial cells in male and female patients with PTC. **e** The molecular networks of the top 100 candidate genes in male and female malignant cells were analyzed. Genes belonging to the corresponding signaling pathways are displayed in different colors (Red represents high enrichment of signaling pathways, and blue represents low enrichment of signaling pathways). **f** Molecular network of Top 100 candidate genes male and female malignant cells; genes belonged to the corresponding signaling pathways and were displayed in different colors. **g** GSEA analysis of representative pathways

CNV is the premise of normal cell cancerization. Epithelial cells from nodular goiter tissues were used as references, and the CNVs of epithelial cells in PTC samples from male and female patients were detected, respectively. The results showed the presence of significant differences in mutation regions and frequencies in both groups (Fig. 2a). Basing on the CNV of epithelial cells, the cell clusters of malignant epithelial cells could be determined (Fig. 2b). Moreover, the distribution of malignant epithelial cells in male and female PTCs showed two different subsets (Fig. 2c). The PCA revealed that the malignant epithelial cells in male and female patients with PTC could be classified into two subsets with obvious differences (Fig. 2d). Furthermore, the gene profiles of malignant epithelial cells were analyzed and characterized in male and female patients. The 50 genes with the highest expression levels were distinctly different between the two groups (Additional file 1: Figure S1). As shown in Additional file 1: Figure S1, multiple members of the *HLA* gene family, including *HLA-DRA*, *HLA-DPB1*, and *HLA-C*, were significantly upregulated in the malignant epithelial cells of female patients with PTC. Additionally, the expression of many genes (such as *RPS4Y1*, *S100A4*, and *SPOCK2*) in male patients with PTC were significantly up-regulated compared with female patients. These results were also highlighted by a ridgeline plot (Additional file 2: Figure S2). The 100 genes with the highest expression were selected for KEGG analysis in malignant epithelial cells, which revealed that the top 10 signaling pathways with the most abundant genes were obviously different between male and female patients. Among them, four pathways (focal adhesion, proteoglycans in cancer, adherens junction, and pathways in cancer) held a dominant position in male patients, whereas an additional three pathways (herpes simplex infection, antigen processing and presentation, and HTLV-1 infection) were outstanding in female patients (Fig. 2e). Concomitantly, the selected genes were analyzed regarding molecular networks. The four most advantaged pathways were significantly different and had almost no intersection between male and female patients (Fig. 2f). Subsequently, the advantaged pathways were subjected to gene set enrichment analysis (GSEA), which showed that the advantaged pathways that played strong positive roles in female patients did not in male patients. Similarly, the advantaged pathways that played strong positive roles in male patients had a weak positive effect in female patients (Fig. 2g).

**Fig. 3:**
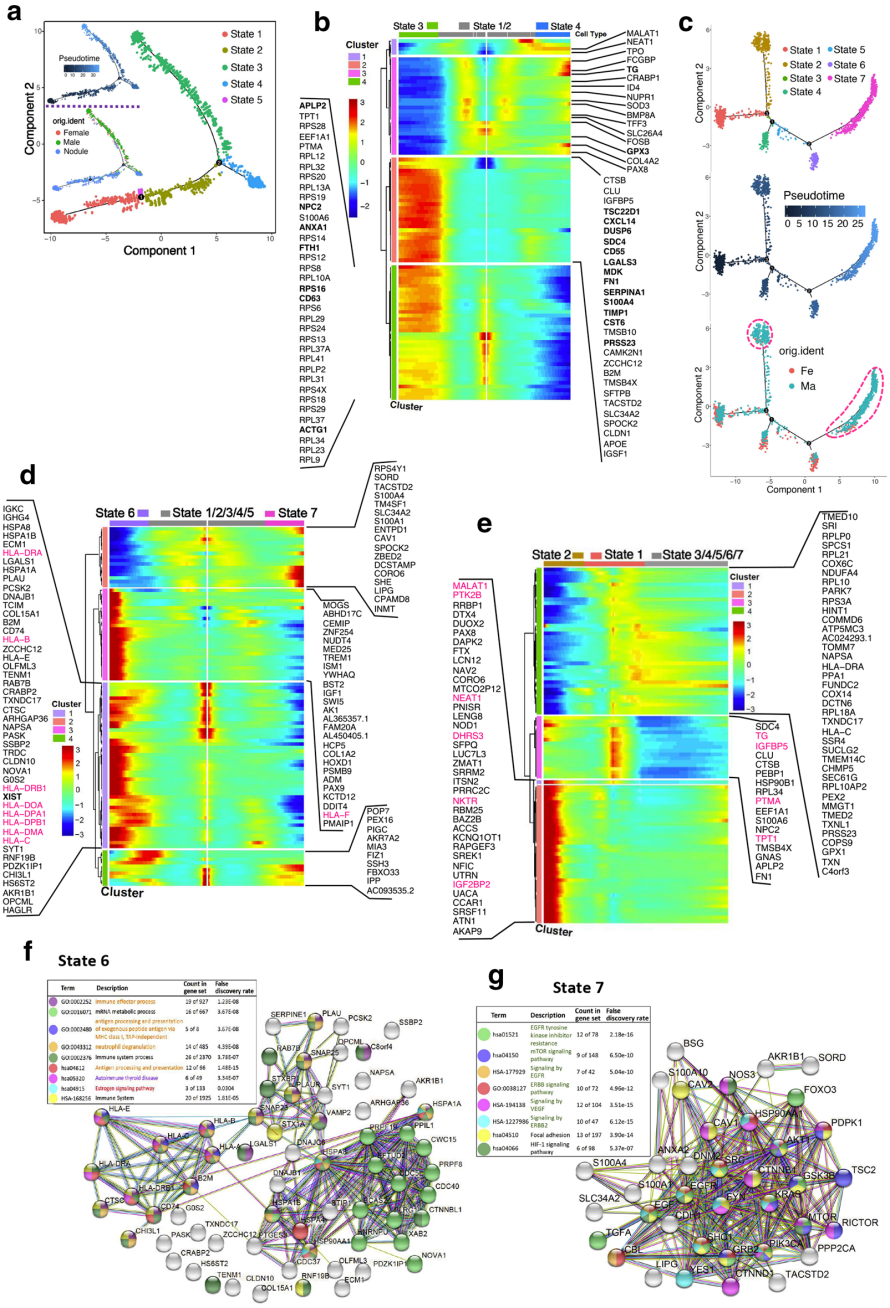
Life process and differentiation of malignant cells in male and female PTC. **a** Pseudotime plot of total epithelial cell clusters of males and females with PTC, analyzed by Monocle. **b** Cluster analysis showing the gene expression profiles of total epithelial cells at different differentiation states. **c** Pseudotime plot of clusters 13 and 31 (Shown in Fig. 1a). Cells from male and female patients with PTC were labeled with different colors. **d **The heat map of Top100 gene with significant difference between state 6 and state 7. **e** The heat map of Top100 gene with significant difference between state 1 and state 2. **f** The pathway enrichment of state 6. **g** The pathway enrichment of state 7

We performed a pseudotime analysis of all clusters of epithelial cells, and defined five differentiation states. Subsequently, we found an obvious aggregation of the malignant epithelial cells in male and female patients with PTC in state 3 (Fig. 3a). Cells in state 1 and state 2 were considered to be in the early stage of differentiation and could differentiate into malignant cells (state 3) and other cell subsets (state 4). The genes related to signal transduction, proliferation, extracellular matrix, and protein synthesis were significantly up-regulated in malignant tumor cells (Fig. 3b). Further analysis showed that the enrichment of malignant tumor cells was greater in male compared with female patients in states 2 and 7, whereas the distribution was uniform in other states according to gender (Fig. 3c). These results were further confirmed the differences in differentiation of malignant tumor cells between male and female PTC group by gene expression profiles and pathway enrichment analysis (Fig. 3d and g and Additional file 3: Figure S3).

In summary, the characteristics of malignant epithelial cells, including gene profiles, cell clusters and cell differentiation, were significantly distinct according to the gender.

### Gender-driven differences in the interaction of malignant epithelial cells with proximal cells in PTC

**Fig. 4:**
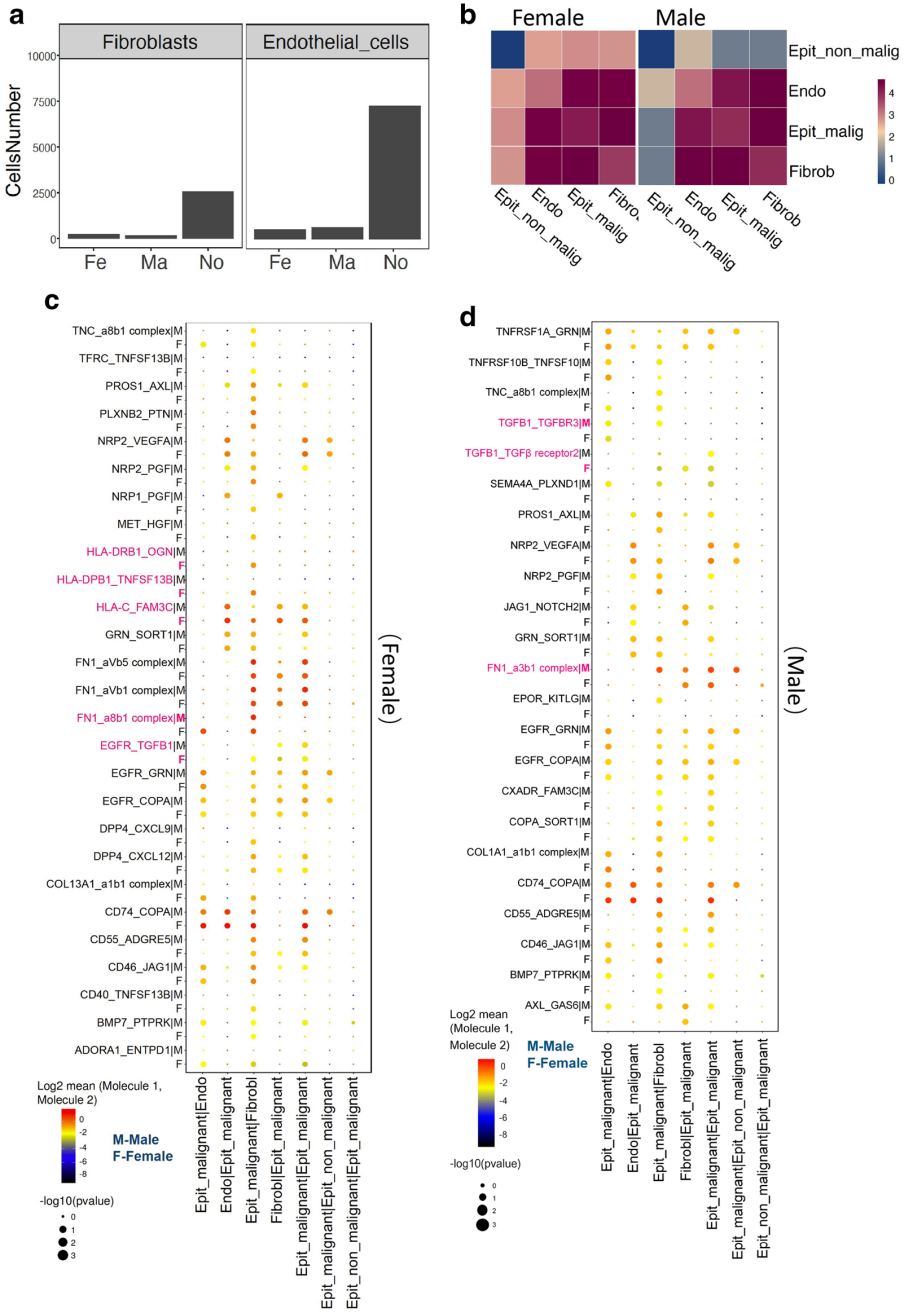
Interactions of malignant tumor cells with fibroblasts and endothelial cells in PTC. **a** Number of fibroblasts and endothelial cells that passed the quality filtering in the three groups. **b** Interactions between four types of cells in female patients and male patients. **c**, **d** The interactions between receptors and ligands in seven different cell clusters in female patients (**c**) and male patients (**d**) were analyzed by Spearman’s correlation coefficient based on gene expression intensity

Several other types of proximal cells, including fibroblasts and endothelial cells, interacted closely with malignant tumor cells and affected their growth, migration, and invasion [10, 11]. Fibroblasts and endothelial cells were less abundant in PTC tissue than the nodule tissue (Fig. 4a). In tumor tissues, malignant tumor cells, ordinary epithelial cells, endothelial cells and fibroblasts were used as the objects of analysis. A cell interaction analysis network was constructed. Obviously, the interactions of malignant epithelial cells with fibroblasts and endothelial cells were strong in both groups (Fig. 4b). Based on ligand-receptor pairs, there were significant differences in the interactions between cells from female and male patients with PTC (Fig. 4c and d).

Taken together, these results suggest that the interactions of malignant epithelial cells with fibroblasts and endothelial cells were different between male and female patients with PTC.

### B lymphocyte infiltration in PTC tissues from male and female patients

**Fig. 5:**
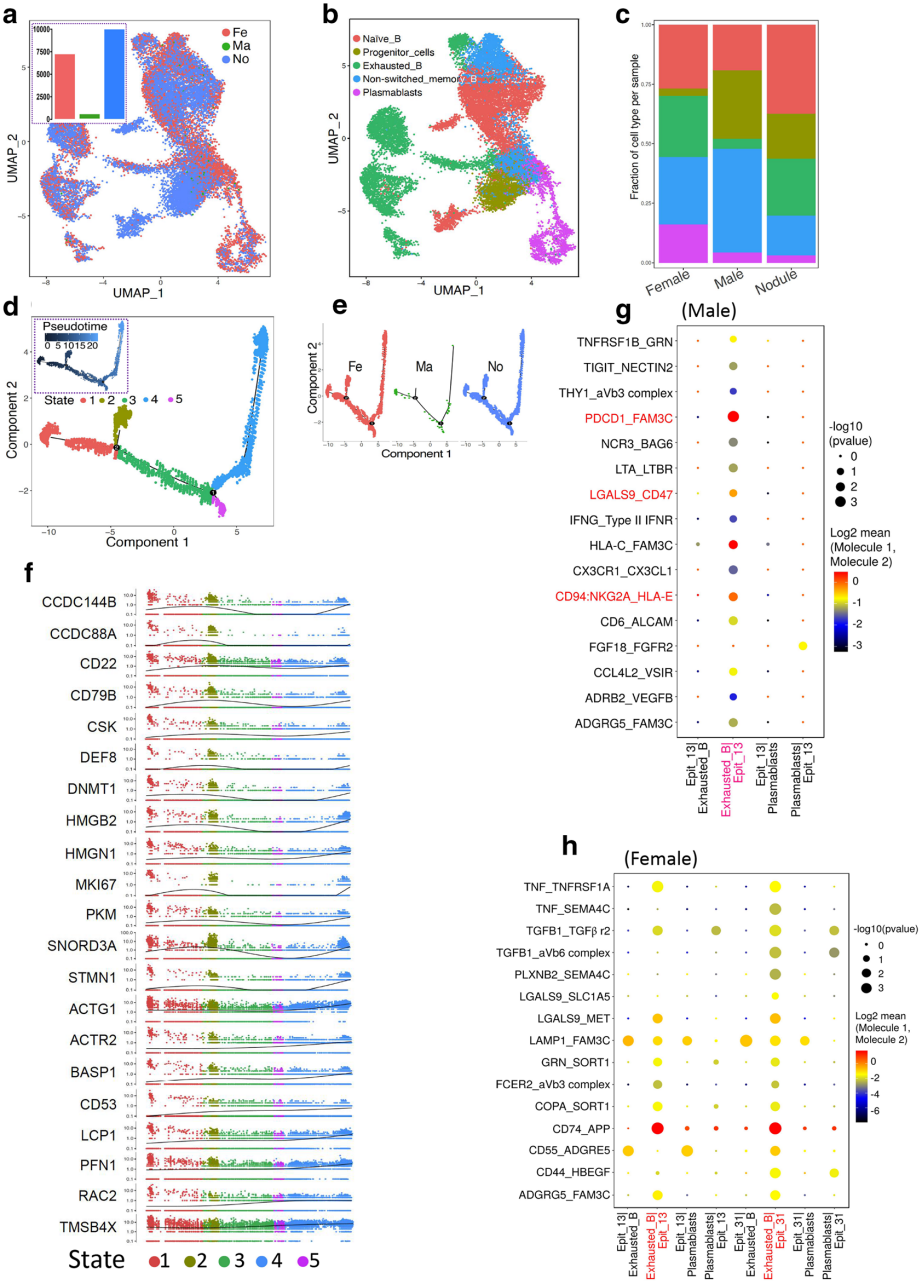
B lymphocyte infiltration in tumor tissues of male and female patients with PTC. **a** Two-dimensional distributions of B lymphocytes in the three groups. The B lymphocytes in different groups are displayed using different colors. **b** Five clusters of B lymphocytes were identified on the UMAP plot. **c** Bar graph of the frequency of the five cell clusters of B lymphocytes in the three groups. **d** Pseudotime plot of total B lymphocyte clusters of male and female patients with PTC. **e** Pseudotime plot of B lymphocytes. Cells from different groups were labeled with different colors. **f** Gene expression of representative genes in states 2 and 4 (shown in **e**) of B lymphocytes. A dot represents a cell, and different clusters are depicted using different colors. **g**, **h** The interactions between receptors and ligands in four cell clusters in male patients (**g**) and female patients (**h**) with PTC were analyzed by Spearman’s correlation coefficient based on gene expression intensity

The infiltration of immune cells into tumor tissues plays an important role in the development and treatment of cancers [12–15]. B lymphocytes were the focus of our uniform manifold approximation and projection (UMAP) analysis. The count of B lymphocytes in PTC from male patients were significantly less than this in other two groups (Fig. 5a). All B lymphocytes in the three groups were clustered into five groups. Among them, it was particularly noteworthy that the proportion of exhausted B cells and plasmablasts was increased significantly in female patients compared with male patients (Fig. 5b and c). The representative genes of the exhausted B cell and plasmablast clusters were analyzed using tSNE and are shown in Additional file 4: Figure S4. The differentiation of B lymphocytes formed two key nodes. Among them, there was almost no differentiation between state 2 and state 4 in male patients, which was obviously different from female patients with PTC and those with nodular goiter (Fig. 5d and e). The typical genes in the differentiation of state 2 and state 4 were screened and displayed in a trajectory distribution of pseudotime, with some overlap with the data in Fig. 5d (Fig. 5f). Based on ligand-receptor pairs, malignant tumor cells (clusters 13 and 31 shown in Fig. 1a), exhausted B cells and plasmablasts were used as the objects of analysis. The results indicated that the interaction involving PDCD1, CD47, and CD94 between cluster 13 and exhausted B cells in male patients was stronger than that observed in female patients with PTC (Fig. 5g and h). In summary, the immune infiltration of B lymphocytes, including cell number, cell differentiation, and the interaction with malignant cells, was significantly different between male and female patients.

### T lymphocyte infiltration in tissues from male and female patients with PTC

**Fig. 6:**
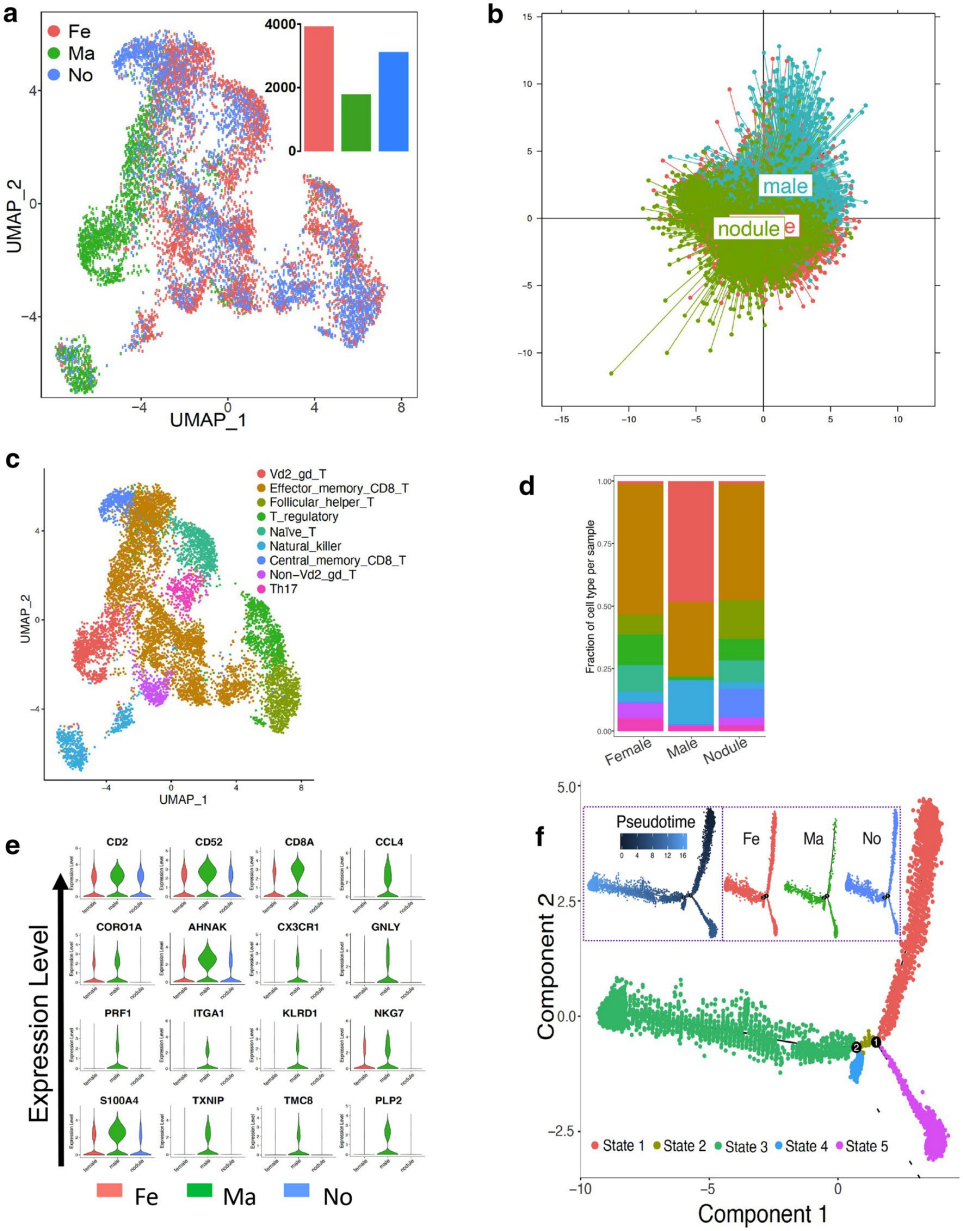
T lymphocyte infiltration tumor tissues from male and female patients with PTC. **a** UMAP plot visualization of T lymphocytes in the three groups. **b** PCA-analysis of T lymphocytes in male PTC patients and female PTC patients. **c** The cell clusters of T lymphocytes are shown using UMAP analysis. **d** Bar graph of the frequency of the cell clusters of T lymphocytes in the three groups. **e** Violin plots of the expression distribution of representative genes of T cell clusters. **f** Pseudotime plot of all T cell clusters

T lymphocytes were assessed by UMAP analysis according to the three experimental groups (Fig. 6a). PCA showed some differences in the differentiation of T lymphocytes between PTC from female and male patients (Fig. 6b). T lymphocytes were clustered in nine subsets (Fig. 6c). Among them, the proportion of three subsets (vd2_gd_T, NK, and CD8 + T) was obviously different between female and male patients (Fig. 6d). It was also confirmed by the expression of the representative genes of the three subsets (Fig. 6e). The differentiation of T lymphocytes formed two key nodes, without significant differences between male and female patients (Fig. 6f).

## Discussion

In the current study, we isolated single cells from PTC (both male and female patients) and nodular goiter samples. We explored the heterogeneity of malignant epithelial cells and the immune microenvironment according to gender using single-cell transcriptomic sequencing. A total of 54,759 cells were captured in three groups, which were classified into eight clusters. There were significant differences in epithelial cells between male and female patients with PTC. Malignant epithelial cells strongly interacted with fibroblasts and endothelial cells. However, their modes of action were different between the two genders. B lymphocyte infiltration in male PTC tissues was significantly less than that in female patients. It was accompanied by differences involving cell differentiation, gene expression profiles, and interactions based on ligand-receptor pairs. These differences were not obvious in T lymphocytes in PTC tissues.

There are significant differences in the incidence rate and prognosis of PTC between male and female patients [1, 4, 6]. CNVs are the basis of the occurrence and deterioration of cancer [7]. Our sequencing results indicated that the dominant mutations of epithelial cells had an obvious gender tendency in PTC, which laid the foundation for the gender heterogeneity of PTC. The TGF-β signaling pathway is deregulated in cancers and can promote cancer cell proliferation [18]. Antigen processing and presentation are notable factors in the efficient immune-mediated elimination of tumor cells [19]. The related signaling pathways may lead to the faster proliferation and poorer prognosis observed in male patients, as well as the weaker migration and infiltration of malignant epithelial cells found in female patients. These results provided an unprecedented explanation at the cell level for the tendency observed among male patients to have a more aggressive disease compared with female patients.

Dysfunction of immune system is associated with the proliferation and metastasis of cancer cells [10, 11]. B lymphocytes play critical roles in producing antibodies and acting as antigen-presenting cells, which have a complicated relationship with cancers [20]. Previous studies showed that tumor-infiltrating B cells were identified as one of the best predictors of outcome and suggested lower relapse rates and increased survival in patients with cervical and lung cancer [21–24]. The number of infiltrating B lymphocytes was obviously lower in male patients, which was consistent with the higher rates of advanced disease stage and worse prognosis. PDCD1, CD47 and CD94 inhibit the killing of cancer cells and are associated with B lymphocytes in many types of tumors [25–27]. Our results indicated that tumor-infiltrating B cells were more significantly inhibited in male patients, including their cell count and immunological function. Meanwhile, we compared the T lymphocytes between male and female patients [28]. T lymphocytes were heterogeneous to a certain extent in patients of both genders, without a fundamental difference.

The present study revealed the detailed differences in cell clusters and immune microenvironment in PTC driven by gender. Despite our major findings, the current research also had some limitations. In general, CNVs in cells are random in the natural state. However, there were significant gender differences in dominant mutations of epithelial cells in PTC. What is the driving force for the accumulation of different CNVs in PTC patients of different genders, hormones or other factor(s)? Based on the analysis of malignant epithelial cells between male and female PTC patients, it is found that there were great differences driving by gender in the trend of cell differentiation, gene expression profiles and signaling pathways, and molecular interaction networks, which would be the potential driving force, finally leading to differences in incidence rate and prognosis between male and female PTC patients. However, their exact mechanism remains unknown. Finally, we found that the cell count and immune functions of B lymphocytes in male patients were more significantly inhibited than those of female patients, which may be potential causes of the poor prognosis of male patients with PTC. In addition, the mechanism of immunosuppression of B lymphocytes remain unknown in male patients with PTC. Meanwhile, there was no obvious difference between male and female PTC patients in T lymphocyte expression. The difference of immune function caused by gender might attribute to hormone levels. Low concentration of estrogen could enhance immune function, while both androgens and high levels of estrogen could suppress immune function [29]. Patients enrolled in the study were mostly young men and young women. Presumably the corresponding levels of androgens and estrogen were at relatively high levels. The suppression of the immune function was potential reason for no significant T lymphocyte expression between male and female PTC patients. Taken together, this was a preliminary study, and additional content will be obtained in future research.

In conclusion, our single-cell results revealed the detailed differences in cell subsets and the immune microenvironment of PTC driven by gender, thus providing new insights for understanding the etiology of the disease and for developing potential therapeutic strategies.

## Supplementary Information

**Additional file 1: Figure S1.** The top50 genes of malignant epithelial cells in male and female patients with PTC, exhibited by heat map.

**Additional file 2: Figure S2.** Ridge plot shown the expression of representative genes which were high-expressed in female with PTC group (left) and male with PTC group (right).

**Additional file 3: Figure S3.** The pathway enrichment of state 1 (A) and 2 (B) of pseudo time series analysis in Fig. 3C.

**Additional file 4: Figure S4.** Expression levels of representative genes in B cell clusters was shown by two-dimensional tSNE visualization.

## Data Availability

All sequencing data generated in this study have been deposited at NCBI’s Gene Expression Omnibus repository, with the accession number of GSE158291.

## Abbreviations

PTC: Papillary thyroid cancer
scRNA-seq: Single-cell RNA-sequencing
CNVs: Copy-number variations
TGFB1: Transforming growth factor beta 1
TIME: The tumor immune microenvironment
DMEM: Dulbecco’s Modified Eagle’s Medium
PCA: Principle component analysis
HLA: Human leukocyte antigen
GSEA: Gene set enrichment analysis
UMAP: Uniform manifold approximation and projection
