# Supplementary figures and images for: Single‐cell transcriptomic landscape reveals the differences in cell differentiation and immune microenvironment of papillary thyroid carcinoma between genders
Source: Cell Biosci. 2021 Feb 15;11:39. doi: 10.1186/s13578-021-00549-w (PMC7885238; doi:10.1186/s13578-021-00549-w)

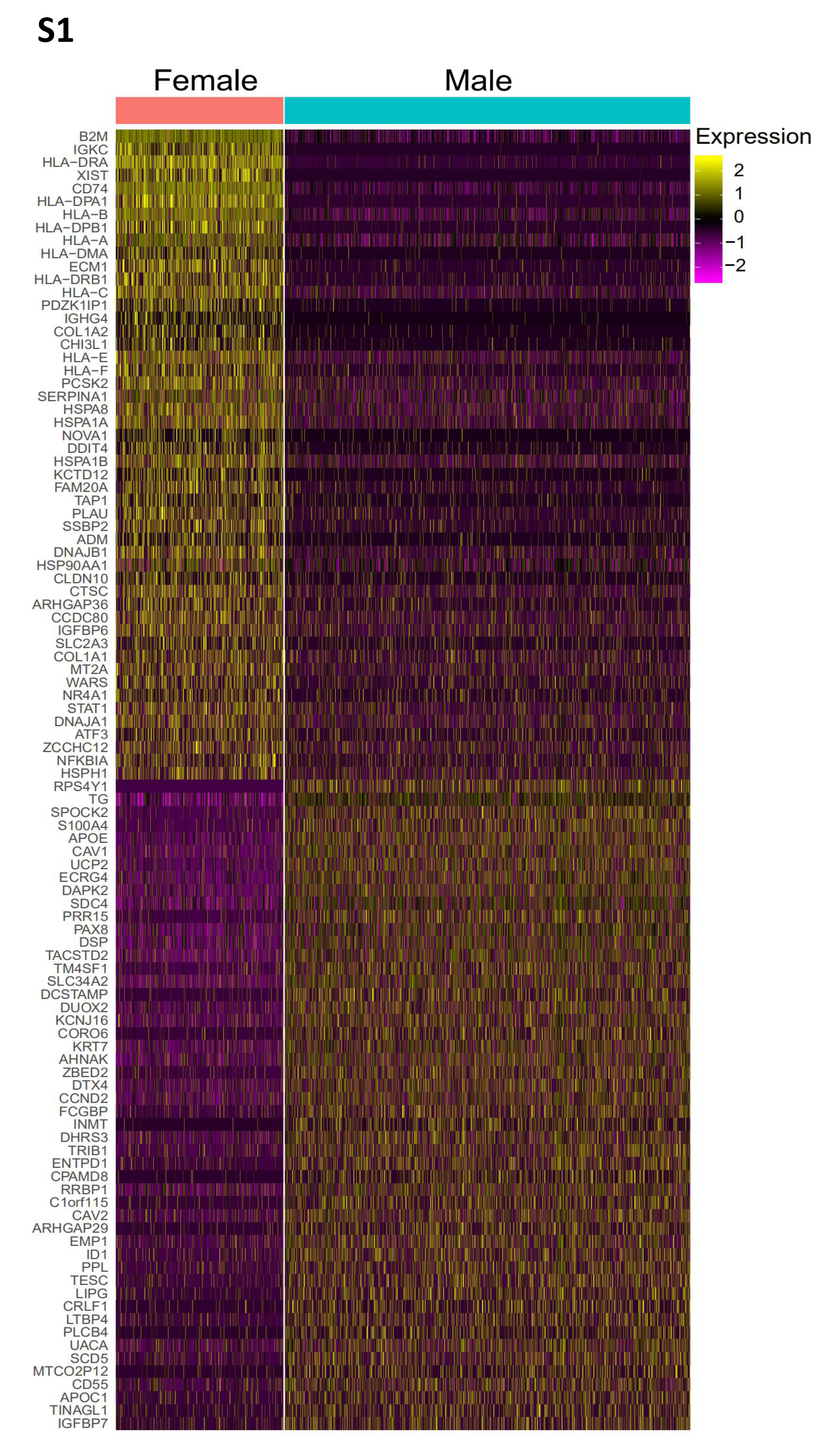

Supplement: Supplementary file 1 — Additional file 1: Figure S1. The top50 genes of malignant epithelial cells in male and female patients with PTC, exhibited by heat map. [file 13578_2021_549_MOESM1_ESM.tif]

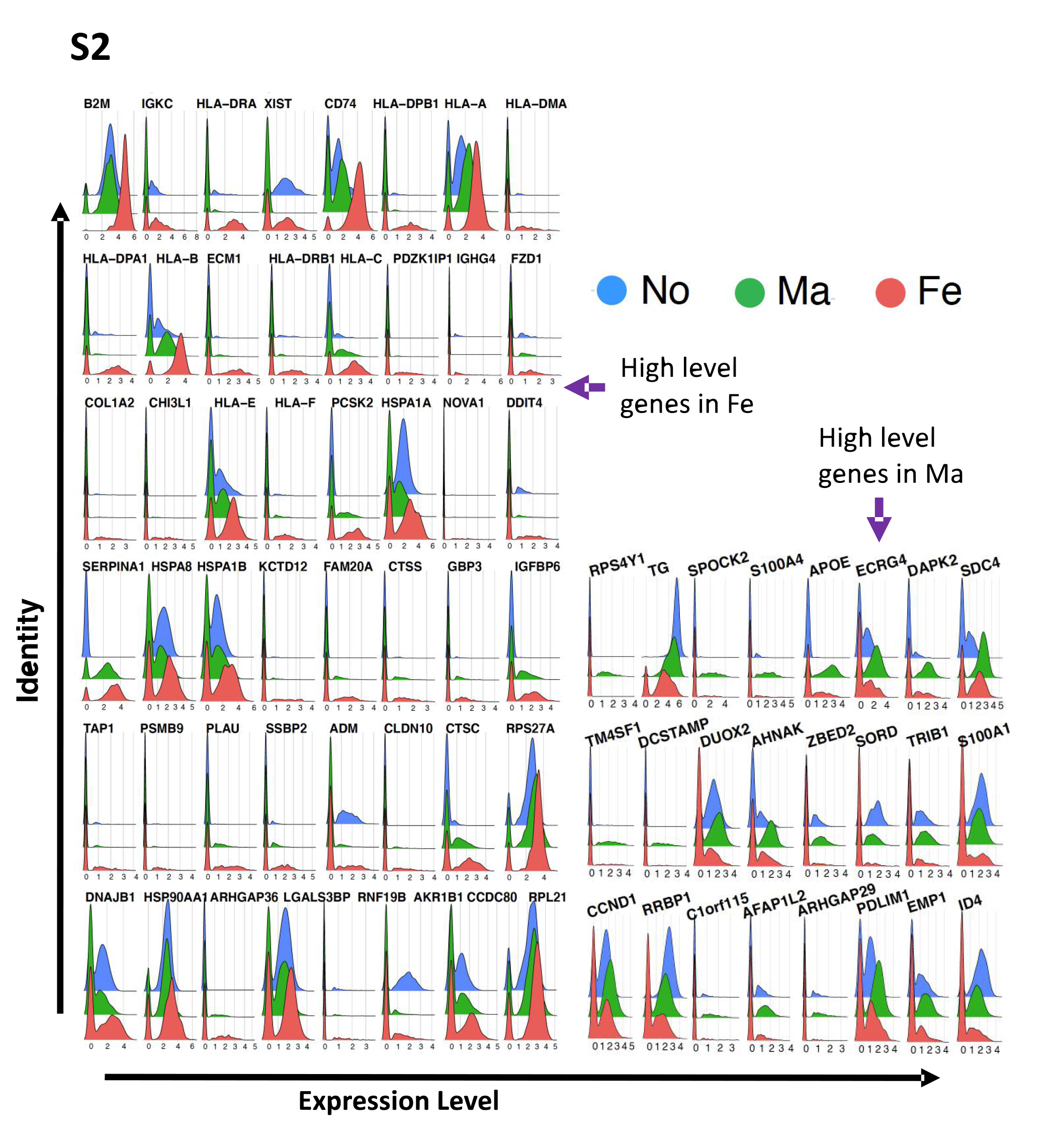

Supplement: Supplementary file 2 — Additional file 2: Figure S2. Ridge plot shown the expression of representative genes which were high-expressed in female with PTC group (left) and male with PTC group (right). [file 13578_2021_549_MOESM2_ESM.tif]

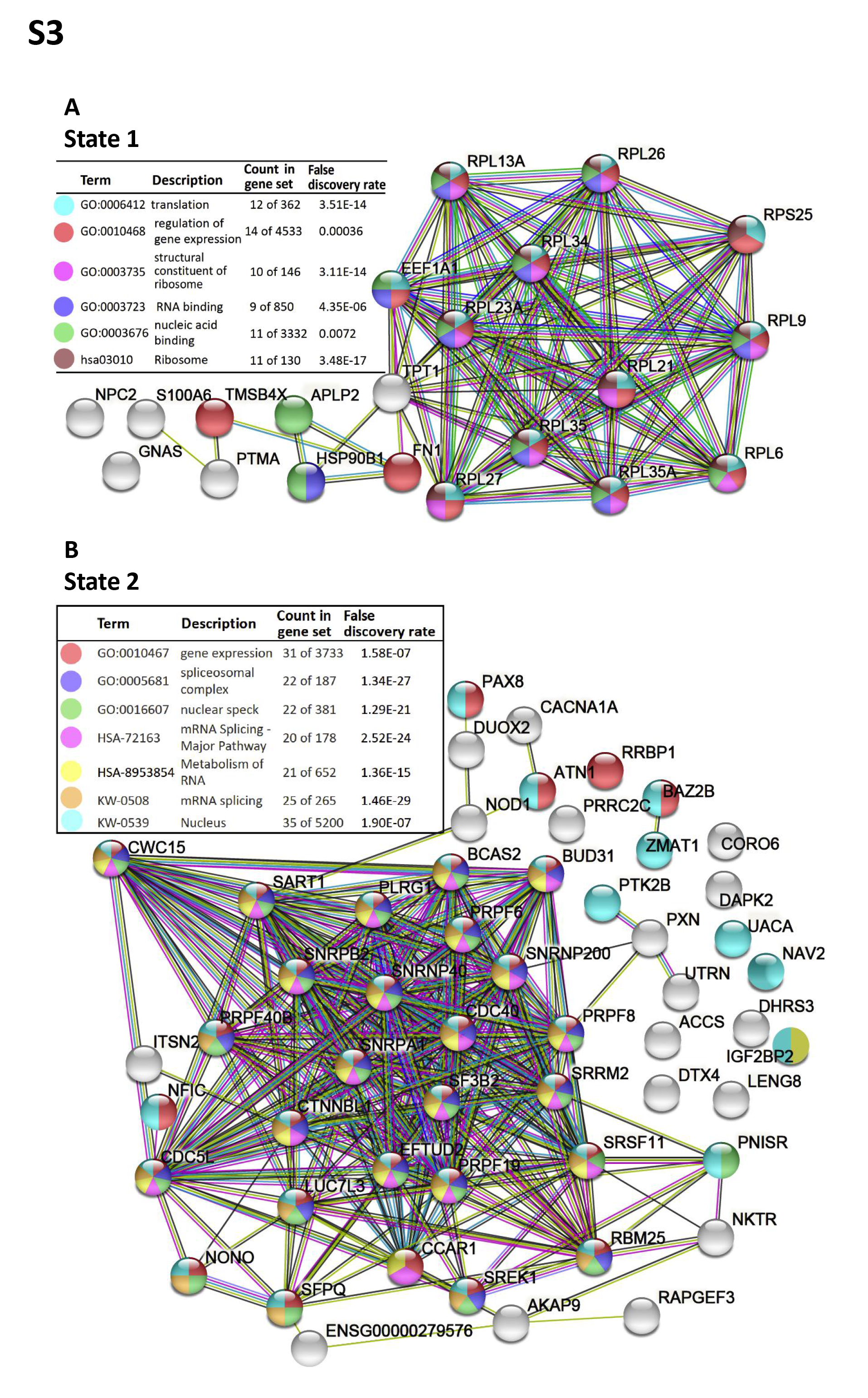

Supplement: Supplementary file 3 — Additional file 3: Figure S3. The pathway enrichment of state 1 (A) and 2 (B) of pseudo time series analysis in Fig. 3C. [file 13578_2021_549_MOESM3_ESM.tif]

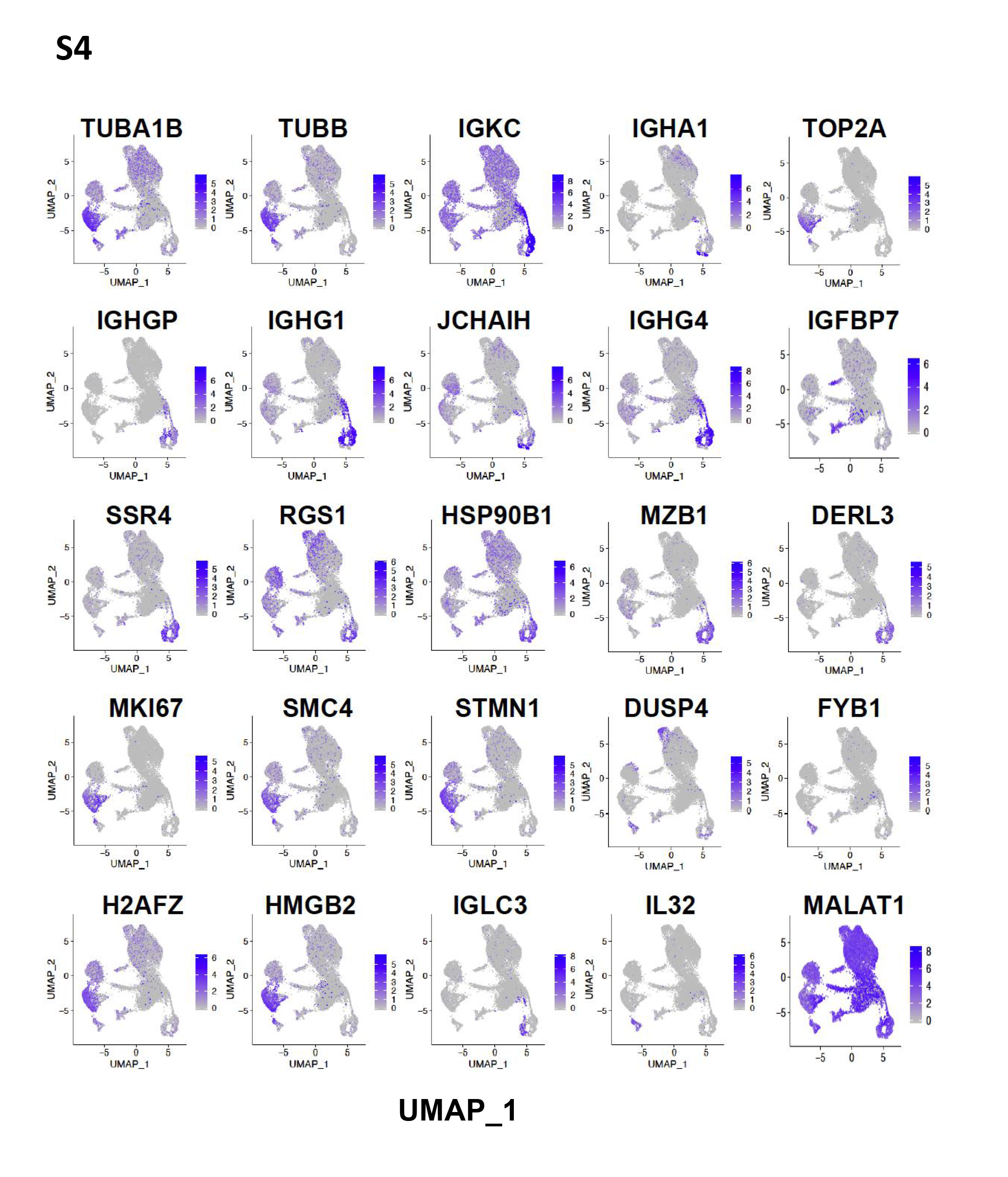

Supplement: Supplementary file 4 — Additional file 4: Figure S4. Expression levels of representative genes in B cell clusters was shown by two-dimensional tSNE visualization. [file 13578_2021_549_MOESM4_ESM.tif]
